# Addressing the Psychological Needs of Adolescents During the Wait Time for Mental Health Treatment: Service Design Study

**DOI:** 10.2196/87067

**Published:** 2026-05-04

**Authors:** Bridianne O'Dea, Roisin McNamara, Ivan CK Ma, Amanda Gu, Fiona Jiang, Justin Milesi, Jeanne Ogilvie, Gywn D Evelyn

**Affiliations:** 1 Flinders University Institute for Mental Health and Wellbeing College of Human Sciences and Culture Flinders University Bedford Park, South Australia Australia; 2 Black Dog Institute Faculty of Medicine and Health UNSW Sydney Randwick, New South Wales Australia; 3 Black Dog Institute Randwick, New South Wales Australia; 4 Deloitte Digital (Australia) Sydney, New South Wales Australia

**Keywords:** design thinking, health service design, mental health care, wait time, service blueprint, lived experience, depression, adolescent

## Abstract

**Background:**

Adolescents waiting for mental health treatment often experience significant unmet psychological needs, including severe psychological distress, increased use of maladaptive coping strategies, and feelings of abandonment. However, current wait time support offerings across the mental health sector are sparse and lack clear evidence of effectiveness.

**Objective:**

Using design thinking, this early report describes the development of a service blueprint for a new model of care (*While We Wait*) designed to address the psychological needs of adolescents during the wait time for mental health treatment in Australia through targeted support from general practitioners (GPs) and brief, self-directed digital interventions.

**Methods:**

In partnership with health service designers from Deloitte Digital Australia, we conducted a rapid 6-week health service design sprint. This industry-led methodology involved iterative weekly activities, including the development of service user personas and service experience principles, consultation sessions with 12 youth with lived experience experts (aged 18 to 20 years) and 15 GPs, insight synthesis, and service blueprint development.

**Results:**

The design sprint produced a service blueprint anchored in 5 service experience principles: “I’m never alone,” “It’s for me,” “I’m in control,” “It’s easy to use,” and “It lifts me up.” The proposed service model incorporated a five-stage service journey: (1) recognition (the adolescent acknowledges the need for support), (2) initial consultation and onboarding with the GP, (3) support and monitoring, (4) preparation for treatment, and (5) transition to specialist care and follow-up. Key adolescent service outcomes included uptake, acceptability, self-advocacy, mental health and well-being, perceived quality of care, and help seeking intentions and behaviors. For GPs, outcomes included uptake, feasibility, acceptability, and confidence in supporting adolescents during the wait time.

**Conclusions:**

This work demonstrates that a rapid, industry-led design thinking approach may help identify priorities for developing services that address adolescents’ needs during the wait time for mental health treatment. The project also highlights the value of co-designing mental health services with lived experience experts and service providers. Together, these findings suggest that the wait time may represent an important opportunity for early therapeutic engagement rather than a passive delay before treatment.

## Introduction

### Background

The wait time for mental health treatment among adolescents is a critical period of unmet need. In Australia, adolescents report waiting more than 100 days to access their first session of treatment after referral by their general practitioner (GP) [1]. Similar wait times have been reported in other regions, including the United Kingdom, the United States, and Canada [2-4]. Modeling indicates that, if current referral patterns persist, wait times for mental health treatment are likely to increase due to limited capacity in mental health care systems to meet demand [5-8]. These prolonged and potentially increasing wait times may exacerbate mental ill-health among adolescents, highlighting the importance of understanding and addressing the impacts of waiting on young people.

Adolescents have reported significant unmet psychological needs during the wait time for mental health treatment, including severe psychological distress, increased maladaptive coping strategies, and feelings of abandonment [1,9]. Long wait times are also associated with treatment dropout, leading to poorer mental health across the life span [9-12]. Despite this heightened risk, wait time offerings for adolescents across the mental health sector are sparse and lack clear evidence of effectiveness for improving mental health and well-being [13-16]. Beyond reducing wait times, mental health services must also be redesigned to ensure that health care providers recognize and respond to the unique needs of adolescents during this period.

### Design Thinking for the Wait Time

Formative research indicates that the wait time for mental health treatment represents a critical gap in service delivery and is often overlooked in service model design despite the significant impact on individuals’ help seeking and overall service experience [17-19]. However, limited research has conceptualized the waiting period as a distinct phase in service flows that warrants specific consideration of consumers’ needs, wants, and interactions. Few studies have explored how to address adolescents’ psychological needs during this period in a timely and responsive manner without adding to overall service demand [14,20,21].

We argue that design thinking (DT) offers a contemporary lens for reimagining the wait time as a unique and critical phase of mental health service delivery. While there is not a uniform definition, DT is generally understood as an iterative, human-centered problem-solving and innovation process that uses methods such as creative thinking, visualization, and experimentation to generate solutions and foster innovation [22,23]. In service design, DT provides a structured, iterative approach that prioritizes the context and needs of consumers and end users, leveraging collaborative multidisciplinary teams with rapid action-oriented ideation to develop solutions [24]. In contrast to empirical scientific practices, DT is a fundamentally subjective practice that facilitates deeper insights into users’ experiences, which encompasses cognitive, affective, and behavioral needs, motivations, and reactions to service events [25,26]. DT offers a process of learning from and together with end users and those with lived experience rather than treating them as research participants. In this way, DT may be particularly valuable in developing services for underserved populations that are underrepresented in traditional scientific research, such as adolescents, and in enhancing complex service pathways associated with high levels of risk, such as mental health care.

It is unclear how widely DT has been applied to mental health service design. A systematic review on DT in health care found only 24 studies using this approach, and only 2 of these were for mental health care [25]. The interventions underpinned by DT were found to have better health outcomes, higher usability, and greater satisfaction than those designed through traditional research methods. Co-design is one method of DT that has increasingly been used to enhance the engagement of adolescents with digital mental health interventions [26,27], with over 20 studies to date applying this approach [28-30]. However, many of these studies have lacked important details on the methods used and rarely involved end users across all stages of intervention design, development, and evaluation [27,28,31]. As such, there are few published applications of DT in the field of adolescent mental health service provision, and none have addressed the wait time.

### Aims of This Project

This early report outlines the application of DT to the development of a new service model (coined *While We Wait*) that aims to address the psychological needs of adolescents (aged 14 to 17 years) during the wait time for mental health treatment in Australia when referred by their treating GP. Specifically, we focused on the application of service blueprinting, which is a flexible process modeling approach within DT that can be used to depict a service, facilitating the refinement of a single step as well as the creation of a comprehensive visual overview of an entire service process. Service blueprints allow all stakeholders to visualize an entire service and its underlying support processes, providing common groups from which critical points of consumer experience can be identified.

Led by service design experts from Deloitte Digital Australia in partnership with young people with lived experience, GPs with professional experience, and digital mental health researchers, this project aimed to develop a service blueprint for supporting adolescents during the wait time for mental health treatment. The blueprint sought to identify the key psychological needs of adolescents during this time; recognize the important role that GPs may play in supporting adolescents while they wait for specialist care; and address these needs through evidence-based approaches delivered via digital technology to enhance accessibility, scalability, and cost-effectiveness. The model was also designed to allow service commissioners the flexibility to adapt or prioritize service components to suit local contexts without compromising the integrity of the core service model.

The initial “essential elements” of the model were identified through a needs assessment phase informed by conversations with youth with lived experience [1], GPs [32], and academic research [21,33] and were subsequently integrated into the blueprint. The model was underpinned by several key assumptions: first, that GPs have the potential to act as trusted advisors for adolescents experiencing mental health difficulties and, second, that young people would be willing to re-engage with their GPs during the wait time for specialist care. These assumptions shaped the design of the proposed service model. However, several design issues needed to be resolved before the model could be operationalized.

This early report aims to describe how we applied DT methods to resolve design issues and refine the service model, with the goal of transforming the waiting period into an opportunity for early intervention and sustained support, ultimately improving mental health outcomes and service accessibility for adolescents.

## Methods

### Design

This project used an industry-led, rapid 6-week health service design sprint, which took place between November 2024 and December 2024. Our reporting of this work aligns with the proposed guideline by Bazzano et al [34] for the reporting of health research involving design.

### Ethical Considerations

The University of New South Wales Human Research Ethics Committee provided ethics approval for the needs assessment among adolescents (HC190382) and GPs (HC220107) conducted prior to this project and published elsewhere [1,32]. The youths with lived experience and GPs involved in the sprint were employed as lived experience and service delivery experts, respectively, and were paid for their time in accordance with the Australian National Mental Health Commission lived experience paid participation policy [35]. Under Australia’s National Health and Medical Research Council National Statement on Ethical Conduct in Human Research 2023 [36], some research is eligible for exemption from ethics review when it carries lower risk to participants or the community, does not collect personal identifiers, and is conducted for quality assurance or program use. We obtained an ethical exemption from Flinders University Human Research Ethics Committee (7902) for this sprint activity as no team members were treated as human research subjects and no personal data collection, testing, or collection of personal materials occurred. All team members were aged ≥18 years.

### Team

#### Operations

A lean operations team was established to coordinate daily activities and consisted of the service design leads (JM and JO) and engagement support (GE) from Deloitte Digital Australia and lead mental health researchers (BOD and RM).

#### Youth With Lived Experience Experts

A total of 12 young people (aged 18 to 20 years) with lived experience (ie, when they were aged 14 to 17 years) of waiting for mental health treatment in Australia co-led the project. Project meetings were structured into a series of small-group interactive workshops to enable active leadership from the youths with lived experience. These meetings were designed to develop and share insights in ways that were age appropriate, engaging, and interactive. Each meeting lasted 1 hour.

#### Service Delivery Experts

A total of 15 GPs with regular experience treating and referring adolescents with mental health problems to specialist treatment or services partnered on the project as service delivery experts. These GPs were engaged with the project from inception and were local to the regions selected for the pilot trial of the service model. Meetings lasted 1 hour for each GP and were conducted separately from the meetings with youth with lived experience.

### Six-Week Design Sprint

#### Week 1: Planning

This phase focused on information exchange between Deloitte Digital Australia and the operations team, facilitated through a series of brief, iterative online meetings held over the week. Expectations and hopes for collaboration were discussed, and all formative research was shared, including research insights from prior studies and user personas. This was used to inform the service designers’ needs assessment, which encompassed their understanding of user preferences and barriers to engagement and exploration of existing mental health services to identify overlaps, gaps, and opportunities. The operations team also prepared the GP and youth with lived experience meetings to confirm the prior needs assessment undertaken by the researchers. This phase allowed for precision in predicting potential user needs, critical interaction points, and existing service gaps, which were later tested and refined in the consultation and mapping phase. Relevant outputs for this phase included a summary of the proposed approach, the proposed timeline, and guiding questions. The service designers prepared weekly status reports on sprint progress.

#### Weeks 2 to 4: Consultation and Mapping

This phase involved iterative meetings with GPs (weeks 2 and 3) and youths with lived experience (weeks 3 and 4), during which we brainstormed service concepts, focusing on accessibility, engagement, and personalization. Where appropriate, ideas were developed into low-fidelity prototypes (ie, early-stage, tangible representations of service concepts), including digital mock-ups such as feedback reports, referral forms, and service flow diagrams. These prototypes were used as discussion tools to explore the feasibility, usability, and acceptability of the proposed ideas. Concepts were refined through several weekly meetings where emerging ideas were iteratively adjusted based on feedback, disagreements were addressed, questions were clarified, and alignment with the project scope was ensured.

#### Weeks 5 to 6: Implementation and Handover of Blueprint

In the final weeks, the service designers shared the final blueprint and provided instructions for handover. The final blueprint was a visual map that included the following sections.

##### Context

This encompassed a brief overview of the need for the service, including who it was for and how it aimed to address the needs of adolescents during the wait time.

##### Service Experience Principles

These provided a clear vision of the desired end-state experience of the service, guiding the creation of consistent, meaningful, and patient-centered interactions. These also established a framework for aligning and evaluating key service interactions.

##### Moments That Mattered

These described the moments across the service journey that were key to effectively meeting the needs of the adolescents and retaining them in the model. Here, the service journey is conceptualized as the full set of interactions, touchpoints, and experiences a consumer has while engaging with a service, from initial contact to resolution [37].

##### User Experience Intent

This was described for each of the key stages, interactions, and experiences. Themes captured from the formal needs assessment and group meetings were used to highlight the critical challenges and opportunities guiding the service development.

##### Service Delivery

This included the “Key Service Interactions,” which were practical descriptions of the key elements of the service that adolescents and GPs would interact with and benefit from during the wait time. The “Process Flow” illustrated the specific steps and interactions within the service, including the key channels and users involved. The “Backend Considerations” were the high-level systems, processes, and capabilities required to deliver the service.

##### Desired Outcomes

The “User Experience Outcomes” represented the intended impacts of the service on adolescents and GPs and provided strategic goals to guide design decisions and a potential framework to evaluate user experience. Additional “Measures of Success” outlined the potential overarching criteria for assessing the effectiveness of the service model, determining whether the service met its goals, and providing a basis for improvement.

## Results

### Overview

The final service design blueprint is provided in Multimedia Appendix 1. On the basis of the needs assessment in the sprint, adolescents were identified as the primary service end users, and GPs were identified as key service actors. Family caregivers, referred service providers, other formal supports (eg, school counselors), and service commissioners were also identified as potential service actors but deemed beyond the scope of the current sprint.

### Service Experience Principles

**Figure 1:**
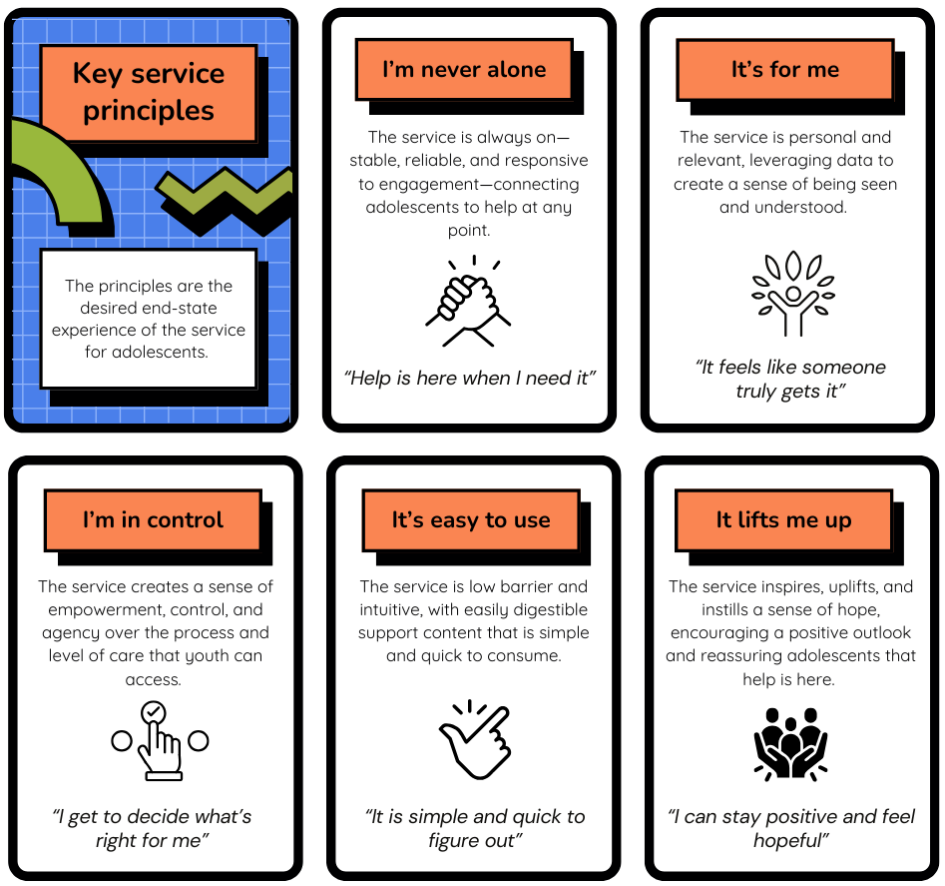
Key service experience principles for adolescents in the While We Wait service model.

For adolescents, 5 key service experience principles were identified to guide the design of support during the wait time: “I’m never alone,” “It’s for me,” “I’m in control,” “It’s easy to use,” and “It lifts me up.” These principles were extracted from the service blueprint (Multimedia Appendix 1) and are explained in Figure 1.

During the initial sprint with youths with lived experience, 2 service experience concepts required further clarification through additional workshopping: personalization and peer support. In relation to personalization, the sprint revealed differing interpretations of the term among stakeholders. To clarify how this concept should be applied within the proposed service model, an additional 1-hour concept-mapping workshop was conducted. During this workshop, youths with lived experience distinguished between different forms of personalization and indicated a preference for support that was tailored specifically to the stage of help seeking (ie, the wait time) rather than to a specific mental health condition or symptom profile (eg, depression or anxiety). It was emphasized that support during the wait time should focus on helping young people manage distress while waiting for care rather than providing condition-specific advice. This preference was reflected in the service experience principle “It’s for me,” which captures the importance of resources and strategies that are tailored to the wait time experience rather than their mental health condition or other situational factors.

The conceptualization of peer support also required further refinement. To explore how peer support could be integrated within the wait time, an additional prioritization workshop was conducted using a prioritization matrix. Existing and potential forms of peer support were mapped, and several features that were considered important during the waiting period were identified. These included access to positive and uplifting stories from peers, support that reflected lived experience perspectives, opportunities to maintain a sense of ongoing connection with others, and opportunities to share their own lived experiences. Youths with lived experience reflected that the existing peer support approaches available to them did not fully align with these priorities for the wait time context.

### Service Journey

**Figure 2:**
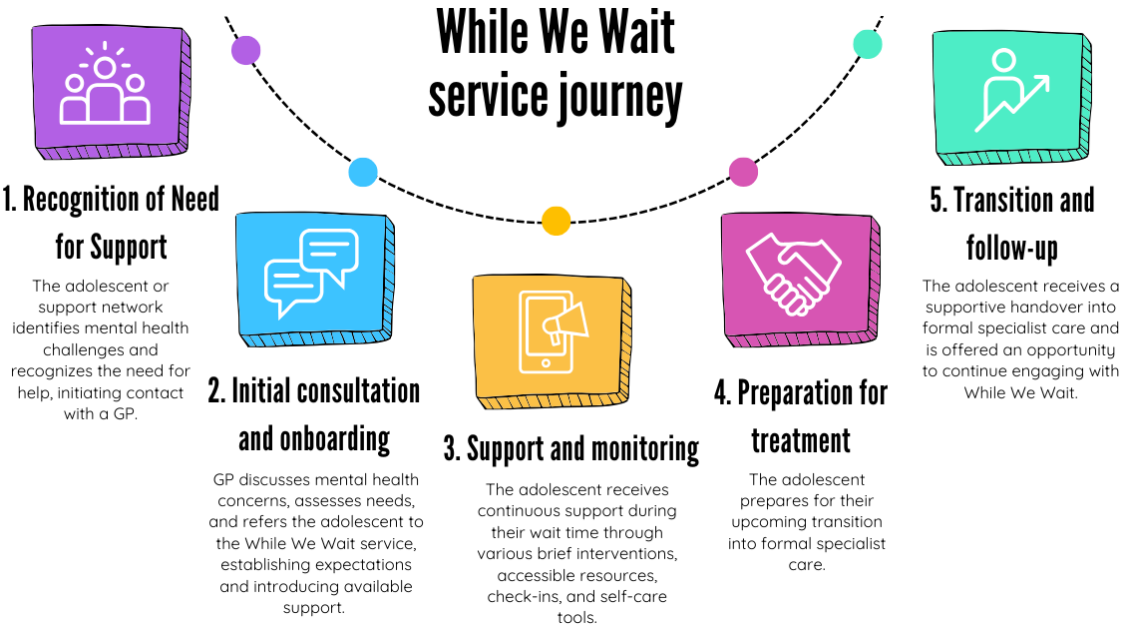
The 5-stage service journey for the While We Wait service model. GP: general practitioner.

Described in Figure 2, the proposed service design encompassed a five-stage journey: (1) recognition of the need for support by the adolescent, (2) initial consultation and onboarding with the GP, (3) support and monitoring, (4) preparation for specialist treatment, and (5) transition to specialist care and follow-up.

**Figure 3:**
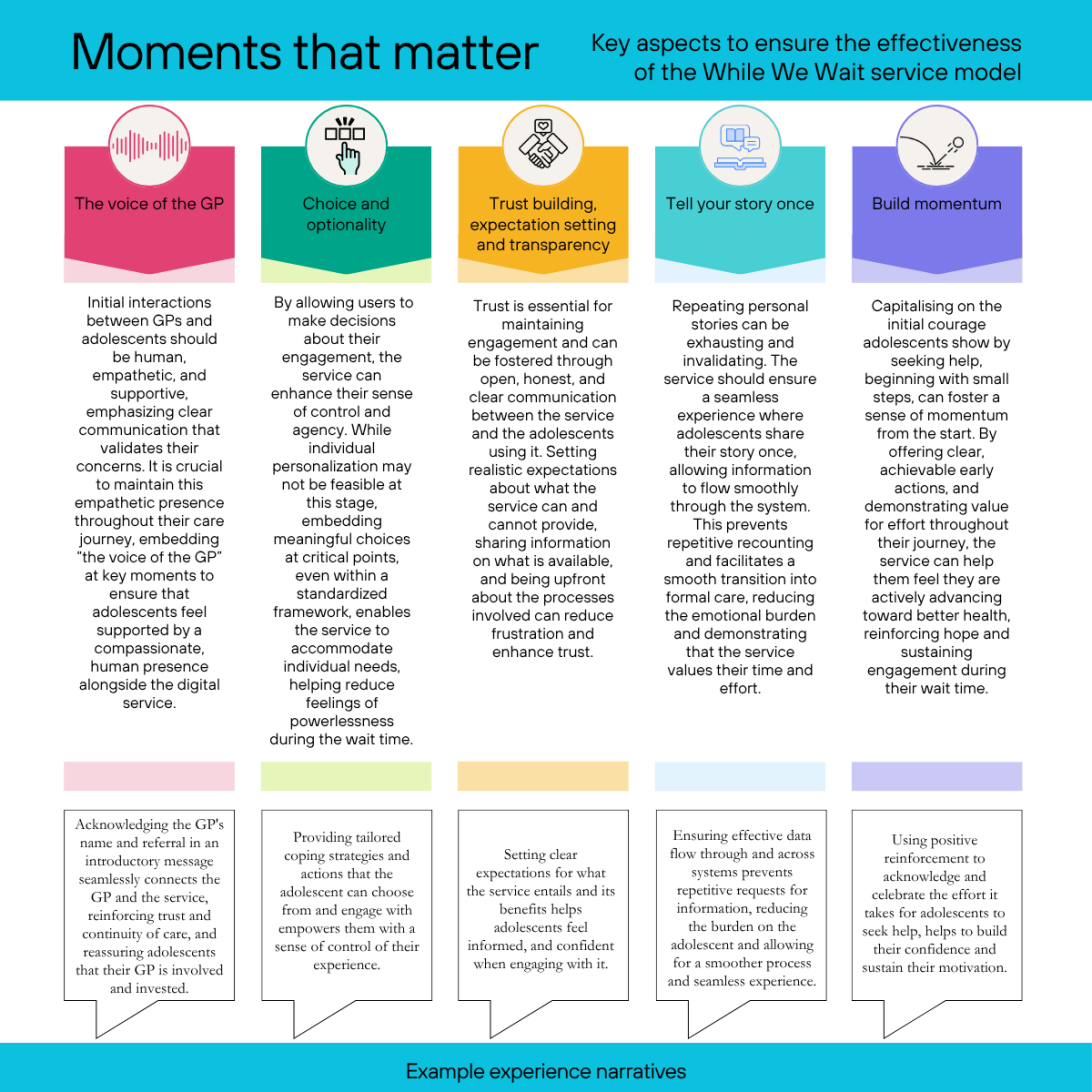
Moments that matter for adolescents in the While We Wait service model. GP: general practitioner.

The sprint also identified 5 key “moments that mattered” in the service flow. Described in Figure 3, these were (1) ensuring that the voice of the GP is empathetic, supportive, and continuously present; (2) enhancing choice and optionality where possible; (3) building trust, setting expectations, and being transparent with adolescents about service features; (4) avoiding repetition and enhancing seamless transition at the beginning and end of the service; and (5) focusing on building momentum in help seeking across each phase of the service journey.

As shown in the blueprint (Multimedia Appendix 1), the core experience outcomes and example measures of success were mapped across the service journey. At the adolescent level, the suggested service outcomes for the pilot trial included their levels of uptake and acceptability, self-advocacy, mental health and well-being, perceptions of the quality of care, and help seeking intentions and behavior. For the GPs, the suggested service outcomes included GP uptake, feasibility, acceptability, and confidence in caring for adolescents during the wait time. Other information on these suggested outcomes is shown in Multimedia Appendix 1.

## Discussion

### Principal Findings

#### Overview

This early report outlines how DT, specifically health service design blueprinting, can be applied to address the psychological needs of adolescents during the wait time for mental health treatment. We provide a practical example of how DT can mobilize interdisciplinary collaboration, bringing together researchers, industry specialists, clinicians, and consumers with lived experience as active contributors to service innovation rather than traditional research participants. We believe that this model of cocreation can provide a more empowering and productive pathway for developing services that are responsive to the needs of adolescents experiencing mental health difficulties. Importantly, the resulting service blueprint illustrates how digital tools, human connection, and ongoing engagement with primary care providers may be combined to transform the waiting period from a passive delay into a potentially therapeutic phase of care. This model provides a foundation for future work to further develop and evaluate wait time interventions for adolescents across different service contexts.

#### Implications for Service Design

A key advantage of DT methodologies is the ability to highlight critical design tensions within complex service models. In our work, three areas emerged as particularly important for further consideration in the design of wait time support: (1) personalization, (2) peer support, and (3) key service actors.

##### Personalization

Personalization is widely discussed in mental health, but its meaning and application can vary substantially across interventions and service delivery contexts [38]. The findings of this project suggest that adolescents conceptualize personalization during the wait time differently from traditional clinical personalization approaches that focus on diagnosis or symptom severity. Instead, adolescents appear to prioritize support that reflects the specific psychological and emotional experience of waiting for care. This distinction highlights the importance of recognizing the waiting period as a distinct phase of service delivery. During the wait time, adolescents may not yet perceive themselves as fully engaged in treatment, which may influence their expectations of support and their willingness to engage with interventions in the interim. As a result, wait time interventions must carefully balance providing meaningful support while acknowledging that adolescents may not yet identify as “being in treatment.” Consequently, adolescents may perceive the waiting period as a “treatment delay” rather than an opportunity for early intervention, highlighting the need for services and interventions to reframe this period. Our findings also highlight the importance of designing services that foster hope and optimism during the wait time. Supporting adolescents to view this time as a meaningful opportunity for support and preparation may enhance engagement and enable other service components to be more effective. As digital technologies continue to advance, particularly with the emergence of large language models capable of delivering adaptive and responsive support, there may be increasing opportunities to provide personalized intervention tailored specifically to this stage of help seeking.

##### Peer Support

Peer support is increasingly integrated into mental health services worldwide, yet its implementation varies considerably across settings [39]. Consistent with prior work [40], the findings of this work suggest that adolescents may value forms of peer support during the wait time that emphasize connection, shared experience, and positive narratives of coping and recovery. This highlights the potential importance of incorporating lived experience perspectives into wait time interventions in ways that foster connection while maintaining appropriate safety and professional support structures. Rather than relying on a single model of peer support, these findings suggest that integrating multiple forms of peer engagement may better address the diverse needs of adolescents during this period. Such hybrid approaches may include structured peer narratives, opportunities for connection with trained peer workers, and other mechanisms that enable adolescents to share and receive lived experience insights in supportive and safe ways. Integrating these modalities within broader service models may help address the social isolation and uncertainty that often characterize the wait time.

##### Service Actors

The importance of GPs as key service actors was consistently reinforced by all stakeholders during the health service sprint. Although the initial service model assumed that GPs would be perceived as trusted advisors by adolescents, many youths with lived experience highlighted the impact of prior negative experiences with some GPs. At the same time, GPs emphasized the importance of actively building a therapeutic alliance with adolescents during the wait time. In response, the service designers increased the prominence of GP-adolescent interactions within the service blueprint beyond what was originally specified in the researchers’ needs assessment. For example, the initial design positioned the service journey as beginning with the first GP consultation. However, insights from the sprint highlighted that adolescents’ preexisting feelings, expectations, and past experiences with GPs critically shape this encounter and must be addressed for the service model to be effective. To address this, we introduced a new help seeking module within one of the proposed automated SMS text message–based digital interventions (*Small Steps*) designed to strengthen adolescents’ trust in their GPs. This module provides practical guidance on how to prepare for appointments and build a more effective therapeutic relationship. We also strengthened the focus on rapport and trust building in the GP training module. In addition, we included an adapted outcome measure to specifically assess the quality of the adolescent-GP relationship [41] and adolescents’ readiness for treatment [42]. Together, these adaptations aim to strengthen the therapeutic alliance between adolescents and GPs, which is critical for improving engagement and outcomes within the service model.

In addition to emphasizing the therapeutic alliance between GPs and adolescent patients, the initial service design blueprint assumed that most adolescents would return to their GPs following an initial referral. While this pattern is commonly reported [1], it may not hold for more disengaged adolescents, representing a potential limitation of the model. The model also assumes that GPs are willing and have sufficient capacity to provide follow-up care during the wait time. Although this level of engagement is typical in adolescent health service use [1], scaling the model will require careful consideration of its impact on GP workload. In the longer term, the model may reduce demand on GPs by addressing adolescents’ needs through targeted, self-directed digital interventions. However, in the short term, it may increase workload, particularly for GPs who have intermittent contact with their adolescent patients. Therefore, collecting data on acceptability and feasibility from both participating GPs and those who decline involvement will be critical for evaluating the long-term viability and success of the model.

The DT approach used in this project was also effective in identifying other key service actors who were integral to the service model but fell beyond the immediate scope of the sprint. Within the current model, family caregivers emerged as a particularly critical stakeholder group requiring further needs assessment. It was recognized that caregivers can play a pivotal role in facilitating adolescents’ engagement with services, supporting retention, and ensuring smooth transitions across different stages of care. However, existing research on parental needs during the waiting period for their children remains limited [43]. Parents have reported experiencing significant emotional burden, guilt, treatment concerns, and fears of the future when seeking help for their adolescent children’s mental health [44]. Preliminary findings suggest variability in their preferences for contact and information during the wait time [45]. In contrast, some adolescents have reported how their parents’ involvement worsened their help seeking by creating barriers to accessing services through refusal of support, lack of shared communication or access to information, and being “too busy” to take them to appointments [46]. Adolescents have also reported that their parents may lack the emotional awareness or capacity to effectively support their mental health and, in some cases, described instances in which their needs for help were not recognized or responded to [47]. In response, our team initiated an additional research study to examine the specific roles and needs of parents during the waiting period, with completion anticipated by the end of 2026. Incorporating parental perspectives on acceptability and feasibility in future pilot studies will be important for further refining the service model. Further investigation is also needed to better understand the roles and needs of other key service actors, particularly referred service providers involved in the transition phase of care. To address this, we have initiated a qualitative study with mental health clinicians and service providers to explore their perspectives on wait time supports. Together, these additional investigations will enable future work to broaden and strengthen the scope of the service model.

### Limitations

While DT offers several advantages, our sprint also highlighted the unique challenges that this approach can pose in mental health care service design, particularly in reconciling differing priorities among stakeholders [24]. Specifically, tensions emerged between adolescents’ perspectives and the priorities of GPs. Some GPs strongly advocated for prioritizing immediate access to face-to-face care (ie, the primary focus should be on reducing wait times overall). In contrast, our youth with lived experience experts expressed greater acceptance of waiting times but emphasized the need for self-management support. This aligns with previous research where consumer satisfaction with care was found to be influenced by subjective perceptions of the wait time rather than actual duration alone [18]. Furthermore, multiple studies have demonstrated persistent discrepancies in service outcome priorities among different stakeholders, including young people and clinicians [47-49]. Thus, a central challenge in applying DT to mental health care lies in balancing the development of services and interventions that are not only effective but also acceptable and feasible for both providers and service users. The iterative nature of the sprint provided the flexibility needed for us to explore stakeholder differences; however, some additional workshops were required, inadvertently increasing resource demands.

The service blueprint was not intended to serve as a comprehensive back-end design document. As such, additional resources will be required to refine the technical specifications and translate the blueprint into a fully operational service. A key consideration in this process will be the level of user effort required to access and engage with the service offerings. The proposed model was centered on SMS text message and web link resources, leveraging the minimal effort needed for user engagement with these modalities. This is an important factor given that many adolescents accessing the service may be experiencing motivational deficits. The sprint enabled the team to propose alternative technical solutions such as smartphone apps or web-based platforms while remaining cognizant of the implementation challenges associated with these modalities [50]. Given the importance of knowledge sharing, disseminating the final technical specifications will be crucial to enabling service providers and researchers to adapt and build upon this model. Additionally, further refinement of the outcome monitoring framework is needed, particularly in selecting appropriate measures that can effectively capture the expected impacts valued by adolescents, GPs, and service designers [51]. Finally, piloting the model within real-world service delivery environments is essential to assess whether the core service principles can be maintained in practice [52].

### Conclusions

This work demonstrates that a rapid, industry-led DT approach may help identify priorities for developing services that address adolescents’ needs during the wait time for mental health treatment. More broadly, the findings suggest that the waiting period for adolescent mental health care should be conceptualized not merely as a delay before treatment but also as a distinct and potentially therapeutic phase of the care pathway. Therefore, DT approaches may offer a promising framework for identifying how services can better support adolescents during this time, particularly when integrating digital interventions and lived experience perspectives. Additionally, the project highlights the value of innovative partnerships between researchers, adolescents with lived experience, industry specialists, and service providers in the co-design of mental health services. Future research should focus on translating these service design principles into scalable interventions and evaluating whether structured wait time supports can reduce distress during the waiting period, improve engagement with care, and enhance subsequent treatment outcomes.

## Appendix

Multimedia Appendix 1 The While We Wait service model blueprint.

## Abbreviations

DT: design thinking
GP: general practitioner

## Data Availability

All data generated or analyzed during this study are included in this published article and its supplementary information files.
