# Supplementary material for: Addressing the Psychological Needs of Adolescents During the Wait Time for Mental Health Treatment: Service Design Study
Source: JMIR Form Res. 2026 May 4;10:e87067. doi: 10.2196/87067 (PMC13184595; doi:10.2196/87067)

While We Wait Service Blueprint

Flinders University | Institute for Mental Health & wellbeing

Version 1.1

Overview

This document outlines the planned service experience for the *While We Wait* initiative, developed in response to an urgent and growing need identified through comprehensive research and collaborative design efforts involving a diverse set of stakeholders. The initiative aims to address the significant and largely unmet psychological needs of Australian youth during the critical wait times for mental health services. Recent research[1] reveals that youth aged 14 to 17 often endure wait times exceeding 100 days, during which they experience severe distress and resort to harmful coping mechanisms due to inadequate interim support.

"While We Wait" aims to bridge this critical gap by introducing an innovative support service for youth and their GPs. Our model delivers multiple low-intensity evidence-based interventions early in a young person's help-seeking journey to generate a positive effect on mental health and treatment-related outcomes, while also offering essential support for GPs to effectively manage and navigate the complexities of interim mental health care.

The Blueprint

This document has been created to illustrate the future state experience for the *While We Wait* service for both young people seeking help and the GPs administering it. The future state experience describes how digital tools, human connection and ongoing GP support can collectively transform a young person's wait time into a productive therapeutic window. These innovations are designed to maintain continuous engagement, deliver support tailored to individual needs, and empower GPs with the insights and tools required for effective mental health management. Specifically, this service aims to provide a scalable and effective solution that facilitates early interventions and continuous support throughout the critical waiting periods.

This future state blueprint is not intended to be used as a detailed backend design document and further analysis and design will be required to understand how best to implement the future state initiatives, concepts, and features captured in this document.

1. Subotic-Kerry M, et al. 2023. medRxiv.doi: 10.1101/2023.08.21.23294348.

Key Concepts

There are a number of key concepts that sit at the foundation of the While We Wait service experience, they are captured in our *Experience Principles* and our *Moments That Matter*.

Experience Principles

These provide a clear vision of the desired end-state experience of our service, guiding the creation of consistent, meaningful, and patient-centred interactions.They also establish a framework for evaluating key service interactions, ensuring that each element aligns with our goal for wait time care.

I'm Never Alone

The service is always on – stable, reliable and responsive to engagement – connecting youth with help at any point.

"Help is always here when I need it. I can count on having support that feels reliable, and ready to connect me with what I need, no matter what's going on."

It's For Me

The service is personal, relevant, leveraging data to create a flexible experience, adaptive to individual needs and creating a sense of being seen and understood.

"It feels like someone truly gets what I'm going through and adapts things to suit my needs. It makes me feel seen, heard, and cared for."

I'm in CONTROL

The service creates a sense of empowerment, control and agency over the process and level of care that youth can access.

"I get to decide what's right for me. I feel confident because I can make choices that help me manage things in challenging times."

It's Easy to Use

The service is low-barrier and intuitive with easily digestible support content that is simple and quick to consume.

"Everything is simple and quick to figure out. I don't have to deal with anything confusing – it's easy to find the help I need when I need it."

It Lifts Me Up

The service inspires, uplifts, and instils a sense of hope, encouraging a positive outlook and reassuring youth that help is on the way.

"I'm able to stay positive, feel hopeful about the future, and remain motivated to do the little things that help me feel better, even when times are tough."

Moments That Matter

These are key touchpoints throughout our service journey that significantly influence how young people perceive and interact with the service. These moments, important for their emotional, functional, or psychological impact, can determine whether young people do or do not continue engaging.

The Voice of The GP

Initial interactions between GPs and young people should be human, empathetic, and supportive, emphasising clear communication that validates their concerns. It's crucial to then maintain this empathetic presence throughout their care journey, embedding 'the voice of the GP' at key moments ensuring that young people feel supported by a compassionate, human presence alongside the digital service.

Choice & Optionality

By allowing users to make decisions about their engagement, the service can enhance their sense of control and agency. While full personalisation may not be feasible at this stage, embedding meaningful choices at critical points, even within a standardised framework, enables the service to accommodate individual needs, helping to reduce feelings of powerlessness during wait times.

Trust Building, Expectation Setting & Transparency

Trust is essential for maintaining engagement and can be fostered through open, honest, and clear communication between the service and the young people using it. Setting realistic expectations about what the service can and cannot provide, sharing information on whats available, and being upfront about the processes involved can reduce frustration and enhance trust.

Tell Your Story Once

Repeating personal stories can be exhausting and invalidating. The service should ensure a seamless experience where youth share their story once, allowing information to flow smoothly through the system. This prevents repetitive recounting and facilitates a smooth transition into formal care, reducing the emotional burden and demonstrating that the service values their time and effort.

Build Momentum

Capitalising on the initial courage young people show by seeking help, beginning with small steps, can foster a sense of momentum from the start. By offering clear, achievable early actions and demonstrating value for effort throughout their journey, the service can help them feel they are actively advancing toward better health, reinforcing hope and sustaining engagement during their wait time.

How to read the Blueprint

1 Project Context	2	4 User experience intent
		5 Service Delivery
		6 Desired Outcomes

2 Blueprint Components

Each of these sections may be broken into individual components as indicated in section 2. Individual components communicate different aspects of the service design or requirements for bringing it to life.

Journey Stages

1. **Recognition of Need for Support** — Young person or support network identify mental health challenges and recognise need for help, initiating contact with a GP.
2. **Initial Consultation & Onboarding** — GP discusses mental health concerns, assesses needs, and refers young person to the *While We Wait* service, establishing expectations and introducing available support.
3. **Support & Monitoring** — Young person receives continuous support during their wait time through various interventions, accessible resources, check-ins, and self-care tools.
4. **Preparation for Treatment** — Young person prepares for their upcoming transition into formal specialist care.
5. **Transition & Follow-Up** — Young person receives a supportive handover into formal specialist care and is offered an opportunity for further engagement with *While We Wait*.

USER EXPERIENCE INTENT

Experience Narrative

This describes the overarching journey of our young people and GPs across the wait time including key stages, interactions and experiences, providing context for the *While We Wait* service design.

Moments that Matter

These describe key moments across the journey that we must get right if we want to create a successful service design that sustains engagement.

Research Insights

These capture the themes from our research that sit behind the service design, and highlight critical challenges and opportunities guiding it's development.

Verbatim quotes have been captured directly from qualitative research involving young people with lived experience and have been edited only for grammatical clarity.

SERVICE DELIVERY

Key Service Interactions (SI)

These provide a practical description of the key elements of the service that our young people and GPs will interact with and benefit from during the wait time.

Process Flow

This illustrates the specific steps and interactions that make up the service, showing how each part connects and flows into the next, indicating key channels and users involved.

Backend Considerations

These are a series of key considerations highlighting systems, processes and capabilities required to deliver the service, and are high-level to be expanded upon through further service development, analysis and detailed design.

DESIRED OUTCOMES

Experience Outcomes

These represent the intended impacts of the service on our young people and GPs, providing strategic goals that guide design decisions and a framework to evaluate the experience.

Measures of Success

These outline the criteria used to assess the effectiveness of the service, helping determine whether it meets its goals and providing a basis for improvement.

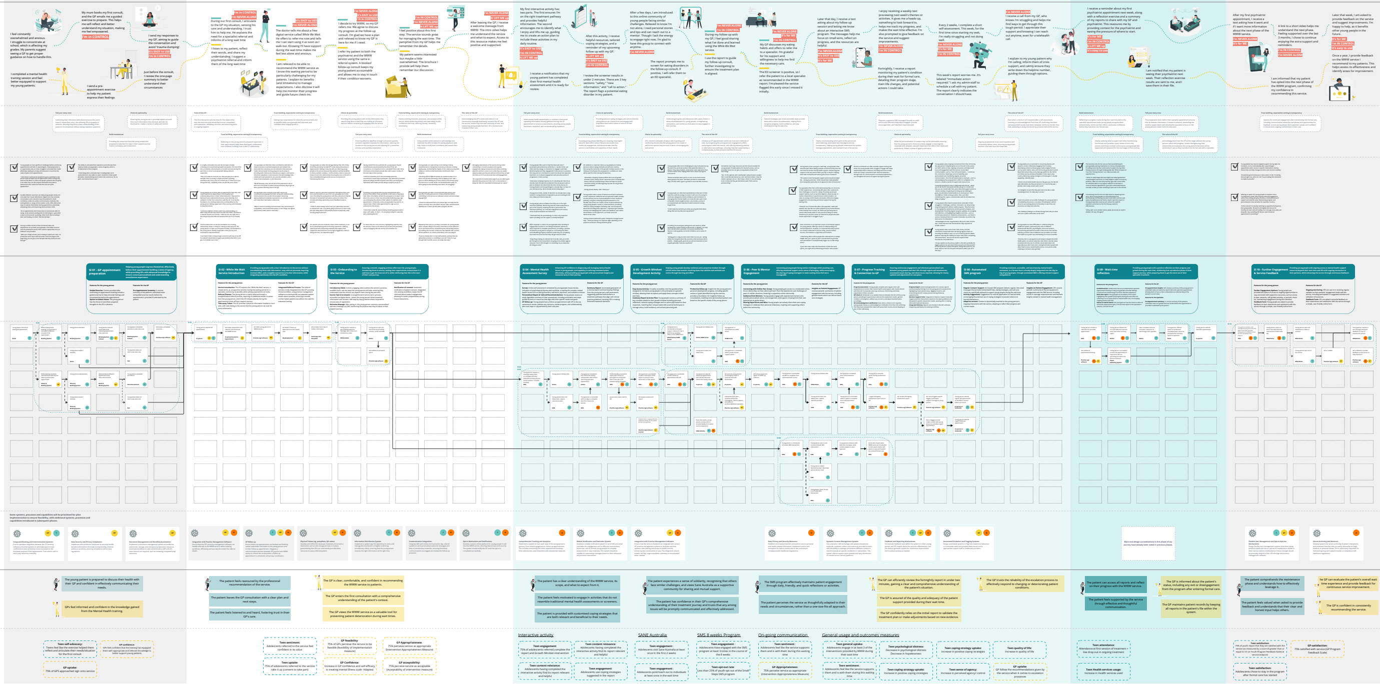

Supplement: Multimedia Appendix 1 [file formative_v10i1e87067_app1.pdf]
